# Factor B Mutation in Monozygotic Twins Discordant for Atypical Hemolytic Uremic Syndrome

**DOI:** 10.1016/j.ekir.2023.02.1069

**Published:** 2023-02-13

**Authors:** Sigridur Sunna Aradottir, Ann-Charlotte Kristoffersson, Brynjar O. Jensson, Patrick Sulem, Henning Gong, Runolfur Palsson, Diana Karpman

**Affiliations:** 1 Department of Pediatrics, Clinical Sciences Lund, Lund University, Lund, Sweden; 2 deCODE genetics/Amgen Inc., Reykjavik, Iceland; 3 Landspitali-The National University Hospital of Iceland, Reykjavik, Iceland; 4 University of Iceland, Reykjavik, Iceland

**Keywords:** atypical hemolytic uremic syndrome, complement, Factor B, genetics, monozygotic twins

## Introduction

Atypical hemolytic uremic syndrome (aHUS) is a life-threatening condition characterized by dysregulation of the alternative pathway of the complement system and manifesting with microangiopathic hemolytic anemia, thrombocytopenia, and acute kidney injury. If left untreated aHUS often leads to kidney failure.^1^ The disease is associated with heterozygous variants in genes encoding complement proteins and regulators including complement factor H, factor I, membrane cofactor protein, (CD46), C3, factor B, and hybrid genes or deletions in factor H-related proteins, or autoantibodies to factor H, leading to uncontrolled alternative complement pathway activation on endothelial cells and thrombotic microangiopathy.^2^ Patients with aHUS may have a family history of the disease^3^ but not all carriers of genetic variants develop disease, even if the phenotype of the mutant variant leads to complement activation. A triggering complement-amplifying event occurring in an individual at risk, possessing one or more genetic variants in alternative complement pathway genes may thereby precipitate aHUS.

In this study, the gain-of-function factor B (*CFB*) variant we previously described, D371G,^4^ was examined in a large Icelandic family with aHUS. The family included monozygotic twins, both of whom are carriers of the rare variant but only one is affected by aHUS. The study traced the ancestral origin of this variant and investigated the phenotype of complement activation in the monozygotic twins discordant for disease expression to further understand the importance of genotype for the development of disease.

## Results

### A Family With Hereditary aHUS

**Figure 1:**
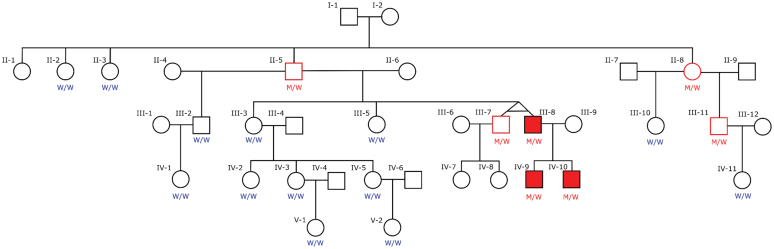
Pedigree of the extended family with atypical hemolytic uremic syndrome. The pedigree shows five generations of the Icelandic family. Individuals whose DNA was tested by Sanger sequencing are marked as “M/W” or “W/W” in which “M” denotes the mutated allele and “W” denotes the wild-type allele. Twenty of the 36 individuals shown were analyzed. The red filled icons indicate the diagnosis of atypical hemolytic uremic syndrome. M, mutated allele; W, wild-type allele.

Three members of one family (see Supplementary Methods) were affected by aHUS. The pedigree is presented in Figure 1 in which individuals III-8, IV-9, and IV-10 are affected. The clinical characteristics and biopsy findings of the affected family members and their treatments are described in Supplementary Table S1. The affected individuals presented with manifestations of aHUS at different ages and experienced a variable disease course.

At presentation, complement levels were available from two of the subjects, III-8 and IV-10, both exhibiting low to low-normal C3 levels (Supplementary Table S2). During remission all three aHUS patients exhibited low C3 levels and normal factor B levels.

### Factor B Variant in the Icelandic Family

The three affected family members were found to have the *CFB* mutation D371G mutation in exon 8 (c.1112 A>G; p.Asp371Gly, ACMG classification^5^: pathogenic). In addition, four family members, unaffected by aHUS, were mutation carriers. Two unaffected carriers (II-5 and III-7, Figure 1) had low C3 levels (data not shown). Two related non-carriers of the *CFB* mutation (III-3 and III-5) had normal C3 levels.

Family members in the extended family (*N* = 203) were screened for the *CFB* variant and were found to be non-carriers. The mutation occurred in the late nineteenth century (in the founder couple of this pedigree or their parents). The mutation was only observed in this family and is absent in 43,445 Icelanders who had whole-genome sequencing data available in the deCODE genetics database.

### Discordant Phenotype in Monozygotic Twins

Individuals III-7 and III-8 are monozygotic twins. The zygosity of the twins was determined by examining common variants (minor allele frequency >5% in GnomAD) in the whole-genome sequencing data. The twins were concordant for all examined variants with high quality calls in both individuals and therefore deemed monozygous.

One of the twins (III-8) developed aHUS while his twin brother is an unaffected carrier of the mutation (III-7). The affected twin experienced a flu-like illness three weeks before developing aHUS. Two decades have passed since twin III-8 presented with aHUS and to-date his twin brother III-7 has not developed any signs or symptoms of aHUS. The twins have a similar body mass index. Further genetic investigation did not reveal *de novo* variants or genomic rearrangements in the affected twin. Samples were not available from the descendants of the unaffected twin to assess their carrier status, and they remain unaffected by disease.

### Sheep Red Blood Cell Hemolysis Induced by Serum From the Monozygotic Twins

**Figure 2:**
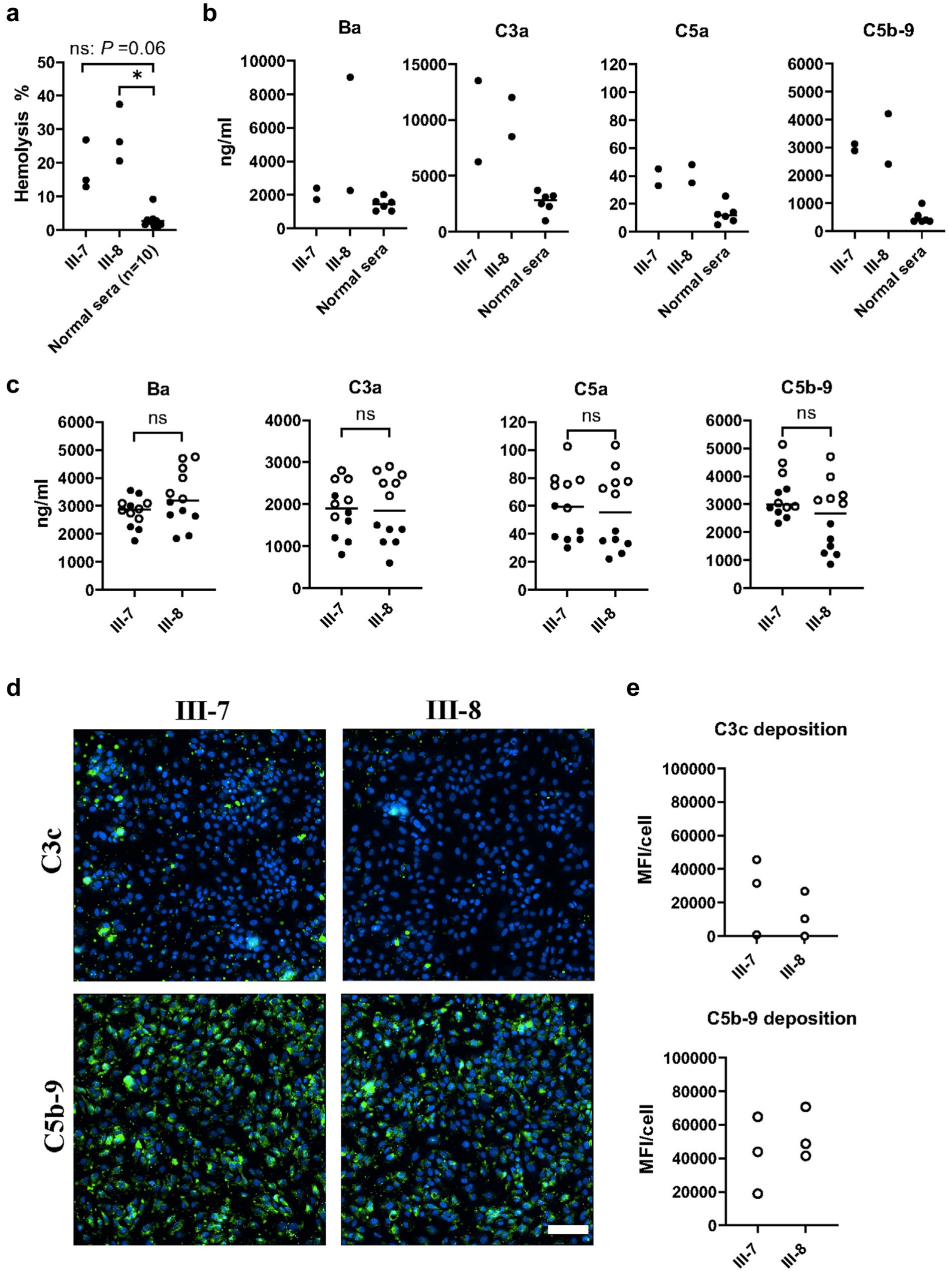
Complement activation mediated by twin sera. (a) Hemolysis of unsensitized sheep erythrocytes incubated with twin (III-7 and III-8, Figure 1) sera obtained at three separate timepoints (2014, 2016, and 2022). Hemolysis is presented as percentage of 100% lysis induced by water. ∗: *P* < 0.05. ns: nonsignificant. Kruskal-Wallis multiple comparisons test followed by Dunn’s procedure. The bar represents the median. (b) Complement degradation products Ba, C3a, C5a and C5b−9 were analyzed in serum samples from two different timepoints (2016 and 2022). Serum Ba was elevated in twin III-8 in a single sample taken shortly after an episode of infection. Complement degradation products C3a, C5a, C5b−9 were found to be at the same level in the sera from both twins and were elevated compared with normal controls (*n* = 6). The bar represents the median. (c) Complement activation was investigated by incubating serum with endothelial cells. Complement degradation product levels were measured in the supernatant from glomerular endothelial cells incubated with sera from the twins. Samples from two timepoints (2016: unfilled circles and 2022: filled circles) were analyzed. For each serum sample, six replicates incubated with cells are presented. Levels of activation products in diluted sera incubated in parallel without cells were subtracted from the presented values. ns: nonsignificant. Mann-Whitney U test. (d) C3c and C5b−9 deposition on glomerular endothelial cells incubated with serum from twins III-7 and III-8. A slight amount of C3c and more C5b−9 were visualized (green labeling). Scale bar: 100 μm. (e) Three replicates of serum incubated with cells were quantified. Fluorescence levels presented in panel B were calculated by measuring mean fluorescence intensity per cell showing an increase in C3c deposition in twin III-7 compared with twin III-8 but no differences regarding formation of C5b−9. Background staining (EGM-2 buffer incubated with cells) was deducted. Statistical comparison was not performed because only samples from 2016 were included. The bar represents the median. MFI, mean fluorescence intensity.

Serum from both the twins increased lysis of sheep erythrocytes and hemolysis was significantly increased using serum from the affected twin (III-8) compared with normal serum. A similar, albeit nonsignificant, trend was noted using serum from the unaffected twin. Results from all three different timepoints of sampling (2014, 2016, and 2022, see Supplementary Methods) are shown in Figure 2a.

### Complement Activation Products in the Serum of the Monozygotic Twins

Serum levels of the complement activation products C3a, C5a, and sC5b−9 were elevated in both twins compared with normal sera (*n* = 6, Figure 2b). Ba levels were not elevated when compared with normal controls except in one sample from the affected twin, III-8, obtained shortly after an infection. A significant difference was not detected when twin sera were compared by multivariate analysis.

### Complement Activation on Glomerular Endothelial Cells Induced by Serum From the Monozygotic Twins

Serum from the twins (from two timepoints) was incubated with primary glomerular endothelial cells and analyzed for complement activation and deposition. Sera from both twins led to detection of complement activation products Ba, C3a, C5a and C5b−9 in the cell supernatants as shown in in Figure 2c. There was no significant difference between the complement activation induced by serum from the affected twin compared with serum from the unaffected twin.

Glomerular endothelial cells were analyzed for C3c and C5b−9 deposition (Figure 2d). Serum from both twins induced C3c and C5b−9 deposition on cells but there was no visible difference between the twin sera regarding complement deposition (Figure 2e). These comparisons were not evaluated for statistical differences because serum from only one time-point was used.

## Discussion

aHUS has been associated with mutations and/or autoantibodies leading to excessive complement activation. aHUS may occur in families, but penetrance is incomplete.^3^ Harboring a disease-associated mutation does not necessarily lead to development of disease. This study investigated a gain-of-function *CFB* mutation, D371G,^4^ in a large Icelandic family in which three family members were affected by aHUS. Within this family, monozygotic twins carrying the *CFB* mutant variant exhibited discordant phenotypes, with one twin remaining unaffected while the other developed aHUS. Furthermore, serum from both twins induced similar complement activation on endothelial cells. These findings suggest that carrying the mutated variant, as such, does not fully account for disease expression but should be considered an important risk factor.

aHUS is not necessarily a monogenic disease because patients may have more than one disease-associated mutation or a risk-associated haplotype.^1^^,^^4^ Conversely, aHUS is not a polygenic disease either because a single mutation may be sufficient for developing disease.^6^ aHUS has been previously described in monozygotic twins,^7^ but not with a discordant phenotype. As monozygotic twins share most genetic variants, the discordance would most probably result from epigenetic variations which may also be linked to environmental exposures such as prenatal and postnatal events, infections, inflammations, vaccinations, as well as lifestyle variations, including body mass index discordance, that may contribute to complement activation and differences between monozygotic twins.^8^

By investigating hundreds of individuals in the extended family, comprising 5 generations, the origin of the mutation could be traced to an ancestral couple born in the late 1800s, or their parents. No other persons were found to carry the mutation in a sample representing close to one-seventh of the entire Icelandic population. Within this family, seven individuals were mutation carriers but only three were affected by aHUS. The aHUS disease phenotype may vary considerably between carriers of the same mutation even in the same family^9^ and the affected individual’s genotype cannot solely explain his or her phenotype, as described herein.

In summary, we describe a large family with a gain-of-function *CFB* mutation and individuals affected by aHUS in 2 generations. In this family, monozygotic twins exhibited a discordant presentation of aHUS despite similar levels of complement activation. We therefore conclude that other nongenetic factors are likely to affect the expression of disease in these individuals.

## Disclosure

The authors have no conflicts of interest to disclose. The authors affiliated with deCODE genetics are employed by the company, which is owned by Amgen, Inc.

## Supplementary Material

**Supplementary Methods.**

**Table S1.** Clinical characteristics of the aHUS patients included in this study.

**Table S2.** Laboratory data of aHUS patients at presentation.
