# Supplementary material for: Factor B Mutation in Monozygotic Twins Discordant for Atypical Hemolytic Uremic Syndrome
Source: Kidney Int Rep. 2023 Feb 13;8(5):1097–101. doi: 10.1016/j.ekir.2023.02.1069 (PMC10166735; doi:10.1016/j.ekir.2023.02.1069)
Supplement: Supplementary File (PDF) [file mmc1.pdf]

Aradottir SS, *et al.*

Factor B mutation in monozygotic twins discordant for atypical hemolytic uremic syndrome

Supplementary methods

Subjects

Three patients with aHUS were diagnosed in the same family (Figure 1 in the main text). One of the family members carrying the gain-of-function factor B variant D371G was previously described.[1] Samples for complement analysis were collected from the three affected family members and from four unaffected family members. Control samples were obtained from apparently healthy adults (n=13, 10 females).

The genotype of an additional 203 relatives was available in the deCODE database. DNA from 210 family members underwent Sanger sequencing. In addition to Sanger sequencing, whole-genome sequencing was performed in 78 of these individuals. Of these, seven individuals are descendants of II-1 and 18 individuals are descendants of II-3 (Figure 1 in the main text). Eleven individuals are second-degree (e.g. niece and nephew) and 61 are third-degree (e.g. grandniece and grandnephew) relatives of a founder male (I-1). Seven individuals are second-degree and 35 are third-degree relatives of the founder female (I-2). Fifty-one individuals are more distant relatives of the founder couple. The database contains whole-genome sequencing data from 45,000 Icelanders. The process of collecting samples and the subsequent imputation of data into the database has been previously described.[2]

The study was conducted with the approval of the Regional Ethics Review Board of Lund University (approval number 2006/323), The Swedish Ethical Review Authority (2021-

04438), the National Bioethics Committee of Iceland and the Icelandic Data Protection Authority. Written informed consent was obtained from all individuals included in the study, patients, healthy controls as well as individuals included in the deCODE database.

Blood samples

Whole blood was drawn into vacutainer tubes according to standard procedure. Samples were centrifuged after one hour to separate the serum, stored at -80°C and, if necessary, transported in dry ice, thawed, aliquoted and frozen at -80°C until assayed. Whole blood in EDTA was transported at room temperature for DNA purification. Samples from two individuals (III-7 and III-8 in Figure 1) were obtained at three separate time-points (in the years 2014, 2016 and 2022).

Genetic analysis and variant screening

Next generation sequencing was performed using Next Seq 500 (Illumina, San Diego, CA) in collaboration with the Center for Molecular Diagnostics, Skåne University Hospital, Lund. The following gene panel was analyzed: *CFH*, *CFHR1-5*, *CFI, MCP*, *CFB*, *C3*, *C5*, *CFP*, *ADAMTS13*, *DGKE*, *PLG*, *THBD* and *CLU*. Whole-genome sequencing was performed on Novaseq (2x150 bp) with mean target depth of 30x. Bioinformatic analysis of variants was performed using Scout software (Similarities from COntinUous Traits https://clinical-genomics.github.io/scout).

Whole-genome sequencing was also performed by deCODE genetics. Paired-end libraries for sequencing were prepared from DNA samples using Illumina preparation kits (TruSeq DNA, TruSeq Nano or TruSeq PCR-Free) according to the manufacturer's instructions. Paired-end sequencing-by-synthesis (SBS) was performed on Illumina sequencers (GAIIx, HiSeq

2000/2500, HiSeq X or NovaSeq) to a target depth of 30x. Read lengths varied from 2 × 76 to 2 × 150 bp, depending on the instrument and/or sequencing kit used. Reads were aligned to the human genome assembly GRCh38 using the Burrows–Wheeler Aligner version 0.7.10.[3] Alignments were merged into a single BAM file and marked for duplicates using Picard 1.117. Only non-duplicate reads were used for the downstream analyses. Variants were called using version 2014.4-2-g9ad6aa8 of the Genome Analysis Toolkit (GATK).[4] The effects of sequence variants were annotated using release 80 of the Variant Effect Predictor (VEP-Ensembl),[5] with RefSeq gene annotations.[6] Sanger sequencing was performed using BigDye Terminator chemistry on a 3730 system (Applied Biosystems, Thermo Fisher Scientific), with primers designed using the Primer 3 software. Data from the large-scale whole-genome sequencing of 43,445 Icelanders was available at deCODE genetics.

Complement biomarkers

C3 and factor B levels were assayed according to hospital routines at the Department of Clinical Immunology and Transfusion Medicine, Skåne University Hospital Lund, Sweden. C3 concentration was assayed by nephelometry and factor B by rocket electrophoresis.

Hemolytic assay

C3 convertase activity leading to hemolysis was assayed by incubation of serum with sheep erythrocytes as previously described.[1] Briefly, serum samples (20%) were combined with equal volumes of normal serum (20%), diluted in gelatin veronal buffer (GVB) containing Mg-EGTA (Complement Technology, Tyler, Texas, USA) and incubated with sheep erythrocytes (Håtunalab, Bro, Sweden). Ethylendiaminetetraacetic acid 10 mM (EDTA, Complement Technology) was added and the samples were centrifuged. The pellet was incubated with rat serum (1:5 in EDTA), as a source of terminal complement pathway proteins, for 1 h at 37°C.

After centrifugation absorbance in the supernatant was measured at 405 nm using Glomax Discover (Promega, Madison, WI).

Complement activation on endothelial cells

Primary glomerular endothelial cells (Cell Systems, Kirkland, WA) were plated on cell culture slides (Thermo Fisher Scientific, Eugene, OR) and grown to confluence. Serum was diluted 1:4 in endothelial growth medium-2 (EGM-2, Lonza, Walkersville, MD). Diluted serum was incubated with (or without) the cells for two h at 37°C. The supernatant was removed and stored at -80°C until analyzed. C3a, C5a, sC5b9 and Ba were measured in the cell supernatant, and in the diluted serum incubated without cells, using commercially available ELISA kits (Quidel, San Diego, CA) according to the manufacturer's instructions.

After removal of the supernatant, cells were washed, fixed with paraformaldehyde 4% and stained for C3c and C5b-9 as previously described[1, 7] with minor modifications. Briefly, after discarding the supernatant, the cells were washed with phosphate buffered saline (PBS) with Mg^{++}/Ca^{++} (GE Life Sciences, Logan) and blocked with 1% Bovine Serum Albumin (BSA, Sigma, St Louis, Missouri) for 1 h at room temperature and fixed. C3c deposition was detected with rabbit anti-human C3c:FITC 1:50 (DAKO, Glostrup, Denmark) in 1% BSA, and sC5b-9 deposition using rabbit anti–human complement C5b-9 1:1000 (Calbiochem, San Diego, CA) followed by Alexa 488-labeled anti-rabbit IgG (green) (Invitrogen #A11070). Nuclear counterstaining was performed with HCD Nuclear mask blue stain (Thermo Fisher Scientific). Slides were stored at -4°C until visualized. Fluorescence was detected using a Ti-E inverted fluorescence microscope equipped with a Nikon structured illumination microscopy module (Nikon Instruments Inc., Tokyo, Japan) and imaged using a Hamamatsu Flash 4 camera and Nikon NIS-elements AR software v.5.11.01. Quantification was performed by Image J Fiji

v2.1.0/1.53c (NIH, Bethesda). Whole well images (10x magnification) were acquired. Triple replicates were performed for each serum sample. Double replicates were performed for the control, i.e., EGM-2 buffer incubated with cells without serum. As the distribution of cells was not uniform in the wells, areas without cells were deducted by choosing the region of interest (ROI) containing confluent cells. The ROI coordinates were marked in the FITC image before quantification. The threshold for fluorescence and area occupied by cells, was set above the background. The mean fluorescent area in each image was quantified as mean fluorescence intensity by multiplying the area with the intensity and dividing by the number of cells. Nonspecific labelling was estimated by quantification of fluorescent labelling in control samples in which the cells were incubated with EGM-2 buffer. This value was subtracted from values calculated for the cells incubated with sera.

Statistics

Comparison between two groups was assessed by two-tailed Mann Whitney U test. For multiple comparisons the Kruskal-Wallis test was performed followed by Dunn's procedure between specific groups. Statistical analysis was performed using Prism software version 9.2.0 (GraphPad, La Jolla, CA).

References

1. Aradottir SS, Kristoffersson AC, Roumenina LT, et al. Factor D inhibition blocks complement activation induced by mutant Factor B associated with atypical hemolytic uremic syndrome and membranoproliferative glomerulonephritis. *Front Immunol.* 2021; 12: 690821.

2. Gudbjartsson DF, Sulem P, Helgason H, et al. Sequence variants from whole genome sequencing a large group of Icelanders. *Sci Data.* 2015; 2: 150011.

3. Li H, Durbin R. Fast and accurate short read alignment with Burrows-Wheeler transform. *Bioinformatics.* 2009; 25: 1754-1760.

4. McKenna A, Hanna M, Banks E, et al. The Genome Analysis Toolkit: a MapReduce framework for analyzing next-generation DNA sequencing data. *Genome Res.* 2010; 20: 1297-1303.

5. McLaren W, Gil L, Hunt SE, et al. The Ensembl Variant Effect Predictor. *Genome Biol.* 2016; 17: 122.

6. O'Leary NA, Wright MW, Brister JR, et al. Reference sequence (RefSeq) database at NCBI: current status, taxonomic expansion, and functional annotation. *Nucleic Acids Res.* 2016; 44: D733-745.

7. Noris M, Galbusera M, Gastoldi S, et al. Dynamics of complement activation in aHUS and how to monitor eculizumab therapy. *Blood.* 2014; 124: 1715-1726.

Table S1: Clinical characteristics of the aHUS patients included in this study

Patient	Sex	Age[a] (yrs)	Clinical manifestations at presentation	Renal biopsy findings	Recurrences	Treatments				
						Plasma exchange	Eculizumab	Kidney replacement therapy	
								Dialysis	Transplantation
III-8	M	41	Malaise, headache, hypertensive crisis[b]	Severe TMA C3	-	+	-	-	-
IV-9	M	24	Dizziness, headache, nausea	NA	-	+	+	+	-
IV-10[c]	M	1	Failure to thrive and irritability	Severe TMA C3	+	+	+	+	x3

a, Age at presentation. b, Three weeks before presentation this patient experienced a flu-like illness. c, This patient was previously described.[1] M: Male. TMA: Thrombotic microangiopathy. NA: Not available. Patient III-8 suffered a single episode of aHUS with severe renal failure that improved markedly following treatment with plasma exchange. One of his two sons (Patient IV-10) developed aHUS with renal failure in early childhood and has since undergone three kidney transplantations[1] while the other son experienced aHUS with severe renal failure requiring transient dialysis, followed by good recovery of kidney function that has since remained stable.

1. Aradottir SS, Kristoffersson AC, Roumenina LT, et al. Factor D inhibition blocks complement activation induced by mutant factor B associated with atypical hemolytic uremic syndrome and membranoproliferative glomerulonephritis. *Front Immunol.* 2021; 12**:** 690821.

Table S2: Laboratory data of aHUS patients at presentation

Patient[a]	Hemoglobin g/L	Platelets $x10^9/L$	LD U/L	Haptoglobin g/L	Creatinine µmol/L	C3 g/L
III-8	83 (130-175)[b]	76 (150-400)[b]	1877 (105-205)[b]	<0.06 (0.35-2.05)[b]	527 (60-100)[b]	0.74 (0.74-0.95)[b]
IV-9	85 (134-171)	77 (150-400)	662 (105-205)	<0.06 (0.35-2.05)	298 (60-100)	NA
IV-10	75 (110-150)	52 (150-400)	1119 (120-600)	<0.06 (0-2.05)	719 (20-40)	0.4 (0.5-0.95)

a, Patient numbering according to Figure 1.
b, Reference values at the clinical laboratory according to the sex and age of the patient at the time of sampling.
LD: Lactate dehydrogenase. NA: Not available.
